# The Effects of Deliberate Rumination and Prolonged Grief on Approach-Avoidance Behavior

**DOI:** 10.1192/j.eurpsy.2025.1959

**Published:** 2025-08-26

**Authors:** Y. Son, C. Lee, M.-H. Hyun

**Affiliations:** 1 Chung-Ang University, Seoul, Korea, Republic Of

## Abstract

**Introduction:**

In the Cognitive-Behavioral Conceptualization of Complicated Grief, avoidance strategies toward bereavement-related stimuli contribute to the development and maintenance of prolonged grief. Traumatic events, such as bereavement, often lead to intrusive negative thoughts and evoke painful emotions. For individuals experiencing prolonged grief, avoidant coping can deteriorate their symptoms. Deliberate rumination on the loss, as an active information-processing strategy to understand and find meaning in the traumatic experience, may promote adaptation to life after the loss.

**Objectives:**

This study aims to examine the impact of prolonged grief on approach-avoidance behaviors and to evaluate the effects of a deliberate rumination intervention.

**Methods:**

Data were collected from 41 Korean adults aged 18 and above, who had experienced bereavement at least 12 months prior. Participants were randomly assigned to either a deliberate rumination intervention group (n=21) or a distraction intervention group (n=20). The Approach-Avoidance Task (AAT) was used to measure implicit approach-avoidance behaviors toward bereavement-related and neutral stimuli in each group, with assessments conducted both before and after the intervention.

**Results:**

The study results revealed no significant differences in approach-avoidance tendencies according to the level of prolonged grief for both bereavement-related and neutral stimuli. However, a significant interaction effect between group and time of measurement was observed for bereavement-related stimuli [*F*(1,39)=4.431, *p*<.05], but not for neutral stimuli [*F*(1,39)=.424, *n.s.*].

**Conclusions:**

Although this study did not identify significant avoidance tendencies according to prolonged grief levels, it experimentally showed that deliberate rumination influences avoidance strategies among individuals experiencing prolonged grief. This finding implies significance in suggesting effective intervention approaches for those with pathological grief.

**Disclosure of Interest:**

None Declared

